# Nanodiamonds as multi-purpose labels for microscopy

**DOI:** 10.1038/s41598-017-00797-2

**Published:** 2017-04-07

**Authors:** S. R. Hemelaar, P. de Boer, M. Chipaux, W. Zuidema, T. Hamoh, F. Perona Martinez, A. Nagl, J. P. Hoogenboom, B. N. G. Giepmans, R. Schirhagl

**Affiliations:** 1 Groningen University, University Medical Center Groningen, Department of Biomedical Engineering, Antonius Deusinglaan 1, 9713 AW Groningen, The Netherlands; 2 Groningen University, University Medical Center Groningen, Department of Cell Biology, Antonius Deusinglaan 1, 9713 AW Groningen, The Netherlands; 3 Delft University of Technology, Dept. Imaging Physics, Lorentzweg 1, 2628 CJ Delft, The Netherlands

## Abstract

Nanodiamonds containing fluorescent nitrogen-vacancy centers are increasingly attracting interest for use as a probe in biological microscopy. This interest stems from (i) strong resistance to photobleaching allowing prolonged fluorescence observation times; (ii) the possibility to excite fluorescence using a focused electron beam (cathodoluminescence; CL) for high-resolution localization; and (iii) the potential use for nanoscale sensing. For all these schemes, the development of versatile molecular labeling using relatively small diamonds is essential. Here, we show the direct targeting of a biological molecule with nanodiamonds as small as 70 nm using a streptavidin conjugation and standard antibody labelling approach. We also show internalization of 40 nm sized nanodiamonds. The fluorescence from the nanodiamonds survives osmium-fixation and plastic embedding making them suited for correlative light and electron microscopy. We show that CL can be observed from epon-embedded nanodiamonds, while surface-exposed nanoparticles also stand out in secondary electron (SE) signal due to the exceptionally high diamond SE yield. Finally, we demonstrate the magnetic read-out using fluorescence from diamonds prior to embedding. Thus, our results firmly establish nanodiamonds containing nitrogen-vacancy centers as unique, versatile probes for combining and correlating different types of microscopy, from fluorescence imaging and magnetometry to ultrastructural investigation using electron microscopy.

## Introduction

In correlative microscopy, a comprehensive view on a specimen is acquired by combining information obtained with different modalities of microscopy. Arguably, correlative light and electron microscopy (CLEM)^1^ constitutes the most widespread form of correlative microscopy. In CLEM, fluorescence microscopy (FM) prior to EM acquisition is used, e.g., to visualize fluorescently labeled molecules within the nano-structural environment imaged with EM. Alternatively one can pinpoint a region of interest for high-resolution EM investigation using live-cell or *in vivo* FM. However, the intrinsic resolution gap between FM and EM limits the degree to which molecules can be localized within the structural EM images. Preferably, this localization would be at the level of EM resolution. A major challenge in CLEM is thus to find approaches and labels that allow live-cell or *in vivo* observation, maintain their fluorescence during EM sample preparation, and can be localized with near-EM resolution.

Direct electron-beam fluorescence excitation, or cathodoluminescence (CL), provides a solution that allows EM localization, but standard organic or biological fluorophores are instable under electron beam exposure^2^. In addition, most fluorescent labels do not survive the sample preparation needed for EM. Colloidal quantum dots are fluorescent, can be used in live cell experiments, and they can be precisely located in EM thanks to their electron dense core^3, 4^. However, CL from bio-conjugated quantum dots, which would allow distinguishing multiple quantum dot labels in color, has not yet been shown. This is probably due to bleaching of quantum dot fluorescence under electron exposure. With phosphor nanoparticles, CL from particles with <50 nm diameter has been observed^5–7^, but application in an EM-prepared sample has to our knowledge not yet been demonstrated. Larger phosphor particles doped with rare-earth atoms have also been explored for upconversion luminescence^8^, which may be attractive in combination with *in vivo* imaging. CL from such particles after cellular uptake and sectioning for EM has been shown^9, 10^. However, so far only particles of >100 nm have been reported, which precludes their use as a molecular label, and conjugation schemes for these particles have not yet been reported.

In recent years diamond nanoparticles containing defect centers have attracted increasing interest^11^ for use as a molecular label because of their excellent photostability. These fluorescent nanodiamonds (FNDs) are also bio-compatible^12, 13^ and can be internalized in cells^14–18^. Further interest in the FNDs stems from the fact that they can be used as local sensors of magnetic^19^ or electric fields^20^, temperature^21^, or strain^22^, which could enable multi-parameter correlative microscopy. Moreover, stable CL from FNDs containing nitrogen-vacancy (NV) centers^5^, as well as silicon-vacancy centers^23^ has been demonstrated, for the NV-FNDs even after cellular uptake and embedding and sectioning for scanning transmission EM^24^ or in live-cell EM studies^25^. However, in these studies the FNDs are large (100–150 nm), limiting the use to cell uptake studies only.

Here, we take the step towards FNDs that are 40 nm and 70 nm in size on average. We show that fluorescence, optically detected magnetic resonance (ODMR), and CL can be recorded from these particles after internalization. Moreover, we present antibody-targeted labelling using the 70 nm FNDs, and demonstrate that these FNDs, targeted to a specific protein can be detected in tissue sections fixed and stained for EM using a standard protocol that allows ultrastructural preservation. Combined with live-cell fluorescence and optical recording of magnetic resonance spectra, our results demonstrate the unique potential of FNDs as biomolecular targets for multi-parameter correlative microscopy.

## Material and Methods

### Nanodiamonds

Fluorescent nanodiamonds of 40 nm (FND40) and 70 nm (FND70) contain 10–15 and >300 NV centres, respectively as stated by the supplier (Adamas Nanotechnologies, NC, USA). FNDs were drop-casted on ITO-coated cover glasses (Optics Balzers, Liechtenstein) and subsequently air-dried. FNDs were analysed with EM using secondary electron (SE) detection for size and dispersity on a FEI Verios scanning EM. The optimal excitation wavelength was assessed using a scanning confocal system (Zeiss LSM 780, Plan-Apochromat 63x/1.40 lens). Using lambda mode, emission spectra were recorded creating intensity profiles between 571 and 687 nm with 9 nm intervals upon excitation with 405, 440, 488, 514, 561 and 594 nm lasers with appropriate beam splitters. Intensities were measured by comparing grey values of the individual images at the 9 nm intervals using Matlab and plotted in arbitrary units. For the size measurements, images were analysed using ImageJ. In total 5051 particles were analysed for FND70, and 1141 for FND40. As the particles were markedly non-spherical^26^, we defined the size as the square root of the surface area of FNDs as detected with secondary electrons. We note that this is an over-estimation as the shortest particle axis will mostly be perpendicular to the surface because of the drop-casting and drying. For the analysis of particle size versus CL intensity, the secondary electron images were used as a mask for the CL signal and the CL intensity of the FND was taken to be the mean signal in the mask areas. To find FNDs with zero CL intensity, the background signal plus one standard deviation was subtracted from the mean CL intensity of each ND, which was normalized afterwards.

### FND_4_0 uptake by J774 macrophages

J774A.1 macrophages (LGC Standards, Germany) which play an important role in the immune system, were cultured in Dulbecco’s Modified Eagle Medium (DMEM) with high glucose, supplemented with 10% FBS, 1% Penicillin/streptomycin and 1% Glutamax (Gibco, ThermoFisher Scientific, The Netherlands). Cells were incubated with 1 ug/ml FND40 in cell culture medium for 5 hours at 37 °C and 5% CO_2_. After removal of culture medium with FNDs, cells were fixed with 4% paraformaldehyde/0.1% glutaraldehyde in 0.1 M cacodylate buffer, pH 7.4 (CaCO) for 30 minutes at room temperature (RT). Nuclei were counterstained with 4′,6-diamidino-2-phenylindole (DAPI). Before optically detected magnetic resonance (ODMR) measurements we used phalloidin-FITC (Sigma-Aldrich, The Netherlands) to label f-actin to visualize the cells. Samples were analyzed using a Zeiss LSM780 confocal microscope using 405 nm and 561 nm excitation. The amount of particles taken up by the cells was estimated using a home written script for the image analysis software FIJI (Fiji Is Just ImageJ, see supplementary information for a detailed explanation). Next the same samples were prepared for EM (see below).

### FND immunolabeling of HT29-EpCAM-GFP cells

HT29 is a human epithelial colon carcinoma cell line, and HT29-EpCAM-GFP stable cells were engineered that overexpress the epithelial cell adhesion molecule (EpCAM) fused to GFP^27^. These were also cultured in DMEM complete medium. Cells were seeded in gamma irradiated 35 mm glass bottom collagen coated dishes (MatTek corporation, MA, USA) until clusters of at least 10 cells grew. Cells were fixed in 4% paraformaldehyde/0.1% glutaraldehyde in 0.1 M cacodylate (15 min, RT) and subsequently blocked in PBS with 5% BSA (PBSA). Then cells were incubated (1 hr RT) with an antibody against the extracellular domain of EpCAM, namely MOC31^27, 28^. After washing in 1% PBSA, samples were incubated with rabbit-anti-mouse-biotin (Dako Netherlands) in 1% PBSA. Cells were washed in 0.1 M cacodylate and incubated with a premixed solution of FNDs and Streptavidin (Sigma-Aldrich, Zwijndrecht, the Netherlands) on a 1:20 weight ratio in 0.1 M cacodylate. As a positive control, cells were incubated with streptavidin-conjugated quantum dots (QD655, Life Technologies, The Netherlands). After washing, cells were imaged using a Zeiss LSM780 confocal microscope using a 488 nm and 561 nm laser. For every sample at least 3 different cell clusters were imaged at 3 different times. Confocal images were analysed and processed (brightness and contrast) using Fiji^29^. EpCAM and FND signal overlap was calculated by manually removing GFP signal coming from membranes inside the cluster using FIJI software. Next the signals were subjected to a threshold and converted into binary values. Next the percentage of FND positive EpCAM pixels was calculated and related to the total of EpCAM positive pixels.

### Sample preparation for integrated light and electron microscopy

We proceeded with the J774 and HT29 EpCAM-GFP cells described above. After washing with 0.1 M cacodylate buffer, cells were incubated with 1% osmiumtetroxide/1.5% potassiumferrocyanide in 0.1 M cacodylate buffer (30 min on ice), followed by washing with water. Next, the cells were dehydrated through an increasing graded ethanol series and left overnight in 1:1 ethanol and Epon (Serva) mixture at room temperature, which was replaced by pure Epon (4 times) and finally polymerized overnight at 58 °C. The cover glass of the imaging dish was removed using hydrogen fluoride. Areas containing cells were selected using a stereo microscope and sawn from the Epon block. Subsequently, 300 nm sections were cut with an ultramicrotome (Leica EM UC7) using a glass knife and put on an ITO coated cover glass. Subsequently, sections were counterstained with Hoechst.

### Integrated light and electron microscopy

Fluorescence preservation was checked using the Zeiss LSM780. Next, the same sections were imaged under high vacuum using a SECOM integrated microscope (Delmic, The Netherlands) in a Zeiss Supra55 scanning EM. The SECOM is equipped with a four color LED, a dichroic mirror (Di01-R405/488/561/635, Semrock, NY, USA), a filter wheel and a CCD camera. Fluorescence of both Hoechst and FNDs was recorded using a 20x/0.75 vacuum compatible objective. EM of the same ROI was acquired using a back scattered electron detector at 10 kV with 60 μm aperture at 9.7 mm working distance. Overlays were created using the SECOM software (Odemis). CL was recorded using a SECOM platform, only equipped with a vacuum compatible plan APO 40x/0.95 light objective and a photomultiplier tube (PMT), retrofitted to a Verios scanning EM (FEI, Eindhoven, The Netherlands). Simultaneously, CL, using the PMT, backscattered electrons, using a circular backscattered electron detector, and secondary electrons, using a through-lens detector, were recorded at either 3 keV and 0.8 nA at a 7 mm working distance. Images were processed and analyzed using Fiji and overlays were created using Adobe Photoshop.

### Optically detected magnetic resonance (ODMR) measurements

**Figure 1:**
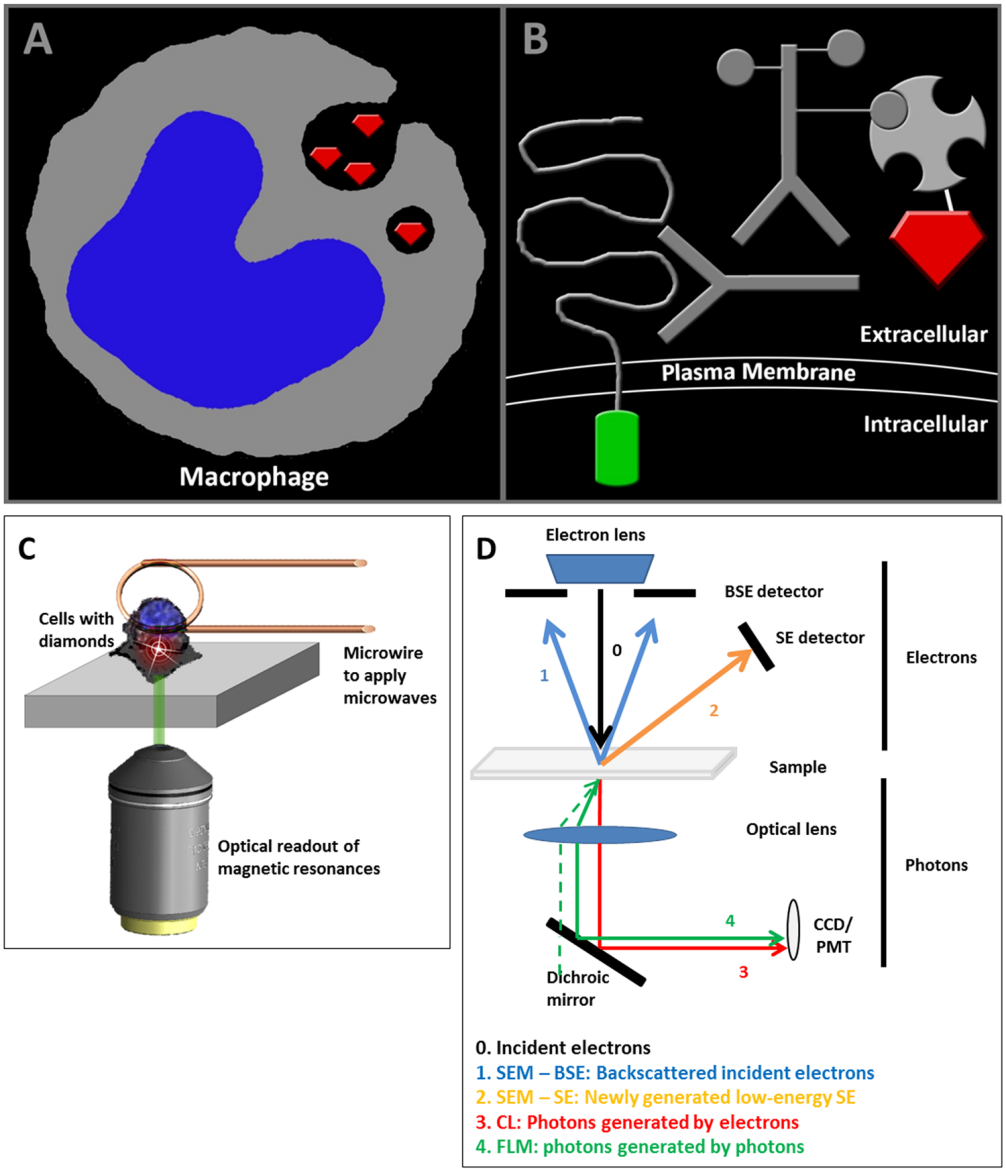
FND applications in this study. (**a**) Representation of FND40 uptake by macrophages. The diamond particles are phagocytosed by the macrophages and are transported in intracellular vesicles. (**b**) Immunolabeling approach: (1) the extracellular domain of EpCAM, with an intracellular GFP domain, is targeted by a monoclonal antibody (MOC-31); (2) biotinylated rabbit anti mouse IgG is used as a linker for labeling with (3) streptavidin-conjugated FND70 particles. (**c**) Schematic overview of the sample area of a diamond magnetometer for intracellular sensing. The cells, which contain diamond nanoparticles, are in a glass bottom petri dish. A microwire in close proximity is used to excite in the microwave regime. Simultaneously, fluorescence is collected through a microscope objective and a subsequent confocal microscope. (**d**) Schematic overview of integrated light and scanning EM and cathodoluminescence. (0) Primary incident electrons generate (1) backscattered electrons (BSE) and (2) secondary electrons (SE) which can be imaged in a SEM with the respective detectors. Also photons can be generated upon electron beam excitation called (3) cathodoluminescence (CL). Via an optical lens, these photons can be detected with for example a CCD camera or photo multiplier tube (PMT). Furthermore, with an integrated light and electron microscope (4) regular fluorescence imaging can be performed with photon excitation.

These measurements allow using the FND to read out their magnetic surrounding. Additionally, they offer a way to undoubtedly identify bright spots as FND defects. For magnetic resonance measurements a home built diamond magnetometer (similar to what is used in the community^30, 31^, see Fig. 1c for a schematic representation), which is a confocal microscope with built-in microwave electronics, has been used. As described previously macrophage cells where stained to identify cell borders. Borders of HT29 cells where identified via their intrinsic GFP signal. To separate the FNDs signal from the other fluorescent staining a 550 nm long pass filter was used. Signal above 550 nm was attributed to the FNDs. A laser power of 1 mW was used. After scanning an area with cells and identifying FND particles we focussed on the FND spots and recorded an optically detected magnetic resonance. The frequency was swept around the expected resonance frequency of the NV centre at 2,87 GHz. This microwave signal was produced with a microwave synthesizer (Hittite HMC-T2100) sending to a homemade antenna (short circuit of a copper wire at the end of a coaxial cable^32^, a few micrometer from the sample). Simultaneously, light intensity was collected using an Olympus UPLSAP40x2 NA = 1,3 objective and an Avalanche photodiode (SPCM-AQRF-15-FC) in single photon counting mode. The microwave power was 27 dBm the acquisition time was 13 min (averaging 300 individual runs).

## Results and Discussions

### Properties of sub-100 nm FNDs

**Figure 2:**
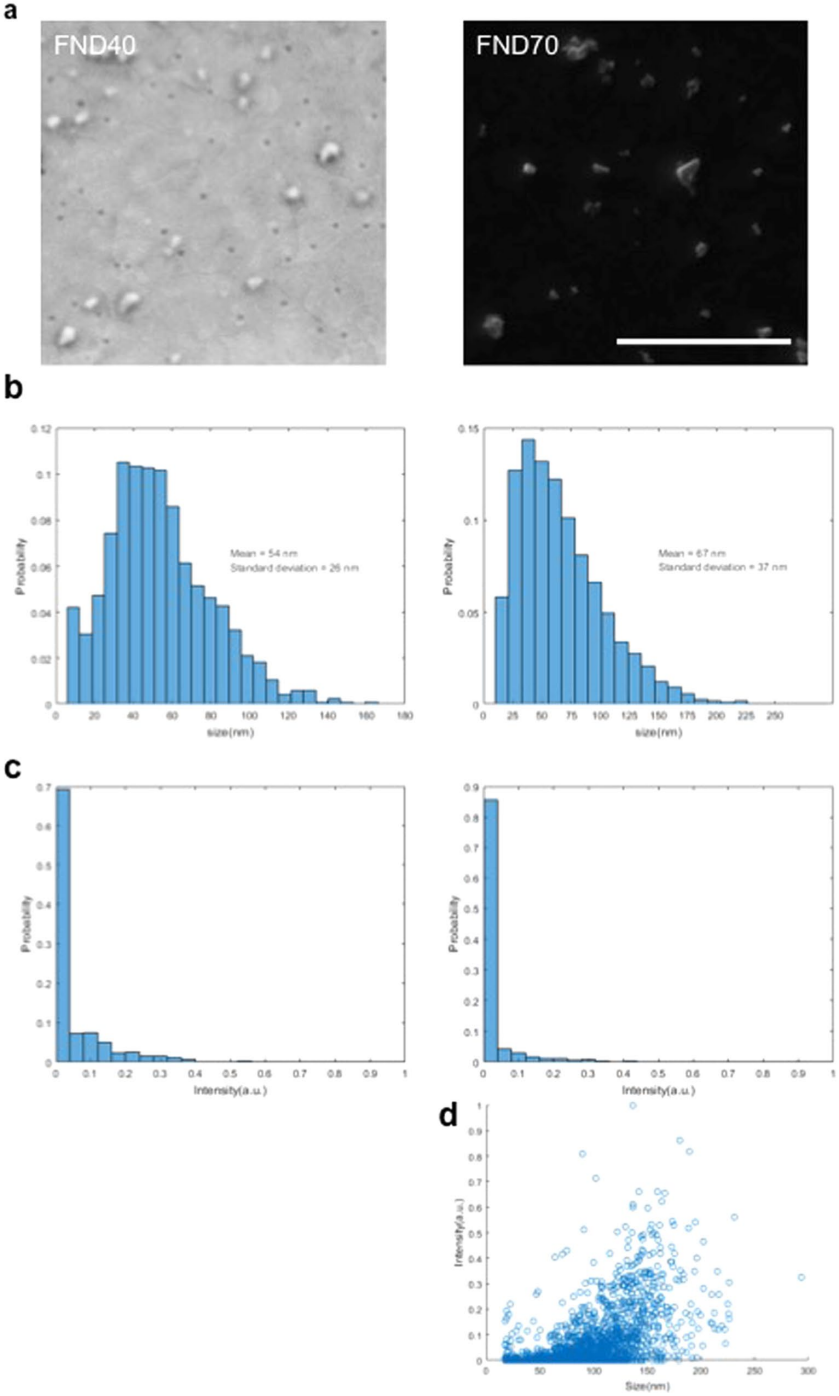
Properties of two different sizes sub 100 nm FNDs. (**a**) Secondary electron images of 40 nm and 70 nm FNDs. (**b**) Size distributions of FND40 (left) and FND70 (right) were defined as the square root of the surface area of the diamonds as detected with secondary electrons as shown in A. (**c**) CL intensity. (**d**) The correlation of diamond size with CL intensity for FND70. Bar: 1 μm.

Recently, attention has gone to relatively large FNDs^33, 34^ which show high fluorescent and CL signals^24^. However, for bio-applications small FNDs, approaching the size of biomolecules, are preferred. First, we set out to characterize the FND40 and FND70 dispersions. When imaged with EM using secondary electron (SE) detection, the different FND types indeed show different sizes when spotted on ITO glass. However, both samples also show a substantial size variation, as can be seen for the FND70 in Fig. 2a. Figure 2b shows the size distributions measured over 5051 and 1141 particles for FND70 and FND40 respectively, which confirm the observed size polydispersity. Distributions are markedly non-Gaussian with average sizes of 54 ± 2 6 nm and 67 ± 37, respectively. Next, fluorescence characteristics were assessed. Excitation with 561 nm laser gave the highest emission intensity, within the red spectrum with a maximum around 660 nm. Furthermore, emission intensity increases with FND size, which can be explained by the higher amount of NV-centres in FND70 (>300) compared to FND40 (10–15). Besides light-excited fluorescence, also electron-excited CL is observed from the FNDs. CL intensity measurements (Fig. 2c) were performed simultaneously with the size measurements. These also displayed strong variations, with a few very bright FNDs and a majority of weak to dim FNDs. Note that fluorescence originates from both neutral (NV°) and negatively charged (NV^−^) vacancy centres, while CL has been reported to only originate from NV° centres. Spectral measurements (see Supplementary Fig. S1) confirmed NV°-only CL. FND size to CL intensity correlation (Fig. 2d) shows that brightest CL originates from the relatively larger FNDs, as may be expected from a larger number of NV-centres in bigger particles together with reduced surface quenching of excitations. However, it is also observed that through the entire size range strong particle-to-particle variations in CL intensity occur. Thus, there are relatively CL-bright small FNDs together with relatively CL-dim larger FNDs. Factors accounting for this may be the unknown out-of-plane diameter of the non-spherical FNDs (although we note that we did not observe correlation between CL and SE intensity), variation in the number of vacancy centres per particle and/or different interparticle ratios of NV° vs NV^−^. Despite the relatively low fluorescence of FND40, the signal/noise measurements as performed on spotted FNDs directed us to evaluate the benefits of smaller size for bio-applications.

### FND40 uptake by macrophages visible with EM and CL

**Figure 3:**
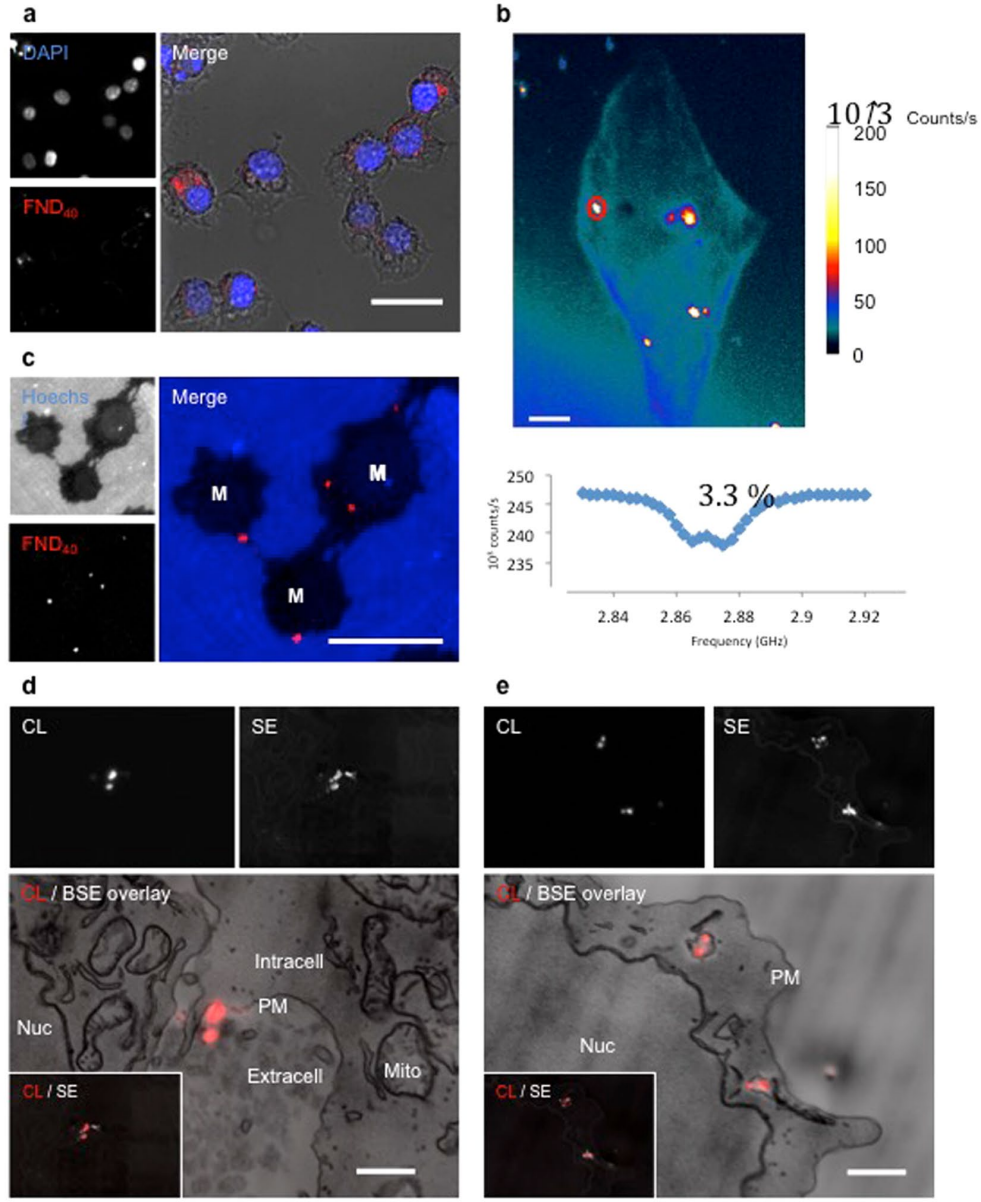
FND40 uptake by macrophages assessed by fluorescence microscopy, EM, magnetic resonance and CL. (**a**) Pre-embedding fluorescence of FND40 internalized by macrophages as seen in the diffraction interference contrast (DIC) merged picture. Nuclei are counterstained with DAPI. (**b**) Magnetic resonance spectra where taken at the bright spots identified as diamonds. The graph shows the spectrum taken at the red circle. 3,3% is the contrast between the resonance line and the background for 1 run. (**c**) Fluorescence from FND40 in a 300 nm semi-thin epon section from macrophages (M). Nuclei are counterstained with Hoechst; blue outside the cells is autofluorescence caused by epon. (**d**,**e**) CL, SE and the CL overlaid with BSE images of FND40 particles. (**d**) extracellular of the plasma membrane during engulfing. The lower left corner shows an overlay of CL and SE of the same image. Some particles are clearly observed with SE and not with CL. (**e**) FND40 particles internalized by the macrophage as they are inside vesicles. The lower left corner shows an overlay of CL and SE of the same image. Some particles are observed with SE and not with CL, but also particles observed with CL are not visible with SE. Note that single FND40 particles within one vesicle can be resolved by CL. M: macrophage; Nuc: nucleus; Intra: intracellular; Extra: extracellular; PM: plasma membrane; Mito: mitochondria; CL: cathodoluminescence; SE: secondary electrons; BSE: Backscattered electrons. Bars: (**a**) 10 μm, (**b**) 12 μm, (**c**), 10 μm, (**d**,**e**) 1 μm.

Recently, FND ingested by different cell types have been shown^9, 10^. We used macrophages for their uptake efficiency as a proof of principle set up for our different imaging approaches (Fig. 1). Indeed, FND40 are taken up by macrophages as assessed by their fluorescence from within the cells (Fig. 3a). Using our home written script for the FIJI software we estimated an average of 210 internalized particles per cell. The identity of the 40 nm diamonds was further confirmed by ODMR measurements revealing the presence of FND40 particles inside the cells (Fig. 3b). The characteristic NV spectrum (a double dip at the resonance frequency of 2.87 GHz) uniquely identifies diamond particles. Next, these macrophages were embedded for subsequent EM. The fluorescence of the FNDs was preserved after conventional epon embedding, including post fixation using 1% osmiumtetroxide (Fig. 3c). The retention of fluorescence could be explained since their NV-centres are embedded in the diamond structure and therefore not accessible for osmium quenching. Loss of fluorescence is often a hurdle in CLEM and maintenance is highly desired especially when an integrated light and electron microscopy approach is used (reviewed in de Boer *et al*.^1^). Although engineered osmium-resistant fluorescent proteins and dedicated embedding protocols for fluorescence preservation exist^35–38^ this may come at the expense of the ultrastructure preservation. Here, however, ultrastructure is preserved, since we used conventional osmium post-fixation and epon embedding (Fig. 3d,e). Fluorescence from the EM samples is however still diffraction limited, precluding precise localization when overlaid with EM data. In order to achieve high resolution localization we utilized the CL properties of the FNDs which have been shown before with single nanodiamonds and for larger FND150 in cells^5, 24, 25^. Clearly, FND40 show up in CL on a dark background (Fig. 3d,e). Variations in CL intensity between different FND40 are observed, e.g., in Fig. 3d where two diamonds stand out and others appear dimmer, likely due to variations in size and number of NV centers contained in the particles. An advantage of SEM is that low energy SEs can be detected simultaneously. As diamond has an exceptionally high SE yield at few keV electron energy, which may even be more pronounced for defected diamond^39^, and SEs can readily escape from the small particles, the FND40 stand out particularly well in the SE image (Fig. 3d). This confirms that the higher CL originates from the relatively larger particles. Compared to the SE image, a halo around the diamonds appears in CL, which we attribute to proximity excitation due to BSE or SE excited in the tissue. When overlaid with backscattered electron (BSE) data we find localisation of FND40 particles within the ultrastructure context of macrophages. This approach reveals different stages of phagocytosis including engulfment as the plasma membrane extrudes around the particles, which are still extracellular (Fig. 3d), and FNDs in phagosomes shown by the presence of a membrane around the particles (Fig. 3e).

### FND70 allow immunolabeling and superresolution detection

**Figure 4:**
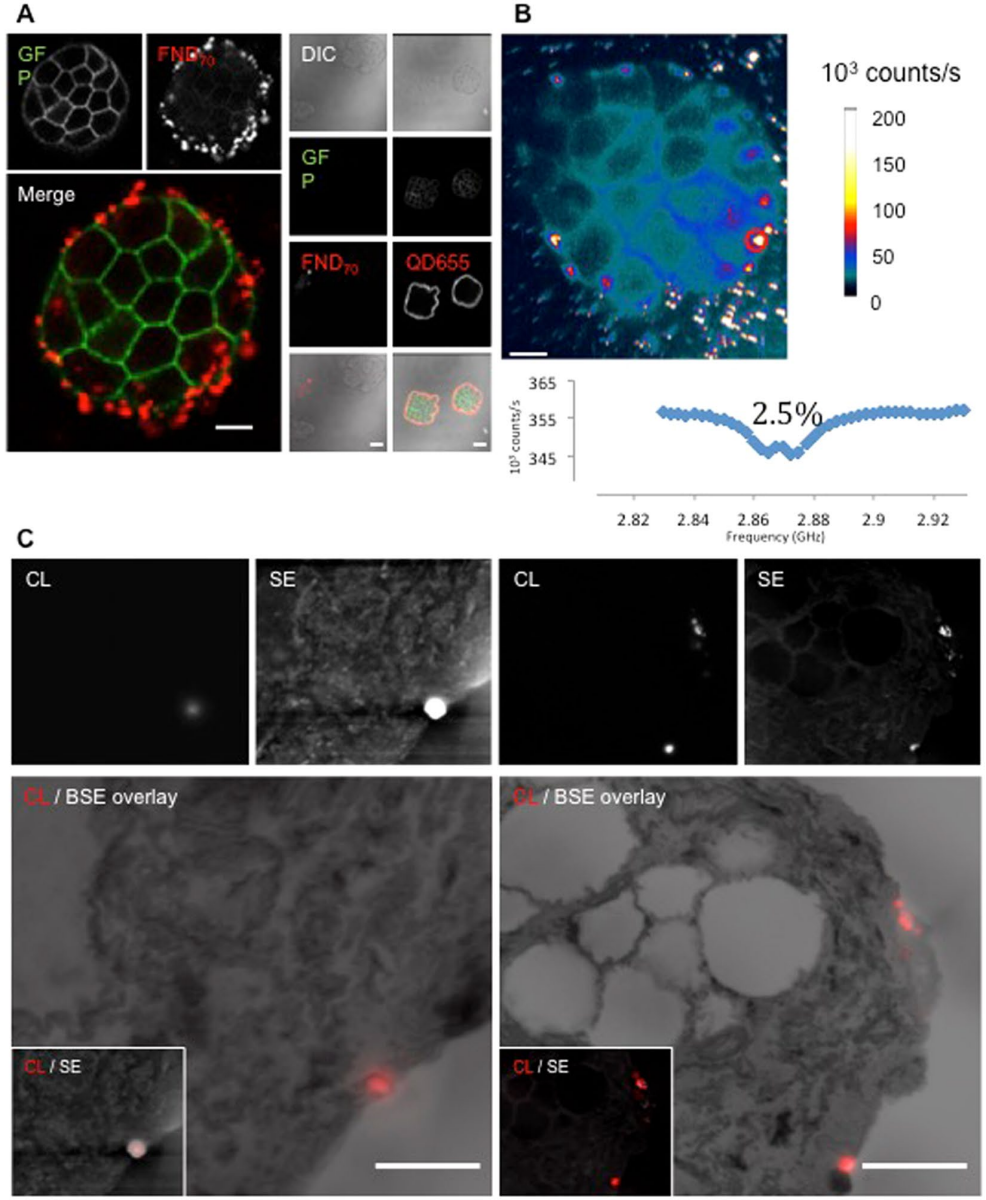
Multimodal analysis of FND70 immunolabeling of EpCAM-GFP HT29 cells. (**A**) Streptavidin-conjugated FND70 labels at the outside of a HT29 cell cluster. The context of different cells within the cluster is shown by GFP. Negative controls (left column) and positive controls (right column; QD655) are shown. No FND70 labeling of non-transfected negative controls is observed, context is shown by diffraction interference contrast (DIC) as EpCAM-GFP is absent. (**B**) Magnetic resonance spectra where taken at the bright spots identified as diamonds. The lower part of the figure shows the spectrum taken at the circled spot. 2,5% is the contrast between the resonance line and the background for 1 run. (**C**) CL, SE and the CL overlaid with BSE images of FND70 labeling an HT29 cell cluster at the cell surface. Note that in the right image single FND70 particles are resolved with CL. Abbreviations as in Fig. 3. Bars: (**A**,**B**) 25 μm, (**C**) 1 μm.

Given the notion that FND40 particles are detectable with CL and EM at high resolution in uptake assays, FND70 was conjugated to streptavidin for generic immunolabeling application. As a proof of principle we immuno-targeted the extracellular domain of EpCAM-GFP expressed by HT29 cells^27^ (Fig. 4A) using a pre-embedding approach without permeabilization to maintain both antigenicity and ultrastructure. Immunolabeling was successful as compared to non-EpCAM-GFP expressing control cells, but sparse compared to smaller QD655 particles (~10 nm). On the other hand, FND70 can be used in ODMR (Fig. 4B). Overlap of the EpCAM positive and FND positive pixels for the cell cluster of Fig. 4A was 22.9%. This imaging mode not only allows proofing that the optical signal comes from diamond particles but also opens up the possibility to measure chemicals or certain properties in the surrounding of the particles via ODMR. Moreover, immunolabeling was detected with CL in EM samples and overlaid with BSE data for high resolution localization (Fig. 4C). Again, FNDs also stand out in SE detection (Fig. 4C). Also very dim or even non-fluorescent nanodiamonds, e.g., due to a lack of CL-active defect centres, show up in SE. On the other hand, only surface exposed particles, would be visible in SE, as for deeper (>few nanometers) lying FNDs, the low energy SEs cannot escape the sample. CL of FNDs for superresolution imaging has mostly been explored using single particles^5, 40^ even with detecting individual defect centres within one nanodiamonds^39^. Here, we show for the first time CL of relatively small-sized FNDs within a biological context (Figs 1d,e and 3c), compared to what others have shown before^24^ to achieve superresolution imaging. Bio-application of CL correlation with EM has been applied before with different nanoparticles, but size is often an issue^9, 10^.

## Conclusions

FNDs have the advantage that they are stable and bleach resistant. They remain fluorescent after osmium fixation, and can also be detected using CL in samples for analysis with EM or integrated microscopes. The CL allows better localization based on the electron beam excitation rather than on the diffraction-limited light detection. Also, FNDs can be used to generate optically detected magnetic resonance signals allowing nanoscale magnetometry. We show proof-of-principle that all these FND properties can be used with small 40 nm and 70 nm particles using uptake assays or immunolabeling. Given the progress made in the last 10 years with using other nanoparticle, e.g., quantum dots, and labels for bioapplications, our results provide a first lead to further develop FNDs for life science research. This might include smaller, differently conjugated or more homogenous particle distributions. Our proof-of-principle of using multi-modal imaging with small FNDs demonstrates (i) fluorescence in EM prepared samples, (ii) CL, (iii) SE and BSD detection, and (iv) magnetometry detection, which will open up possibilities to gain additional information on the magnetic surrounding of the particles.

## Electronic supplementary material

Supplementary info
