# Supplementary material for: Nanodiamonds as multi-purpose labels for microscopy
Source: Sci Rep. 2017 Apr 7;7:720. doi: 10.1038/s41598-017-00797-2 (PMC5429637; doi:10.1038/s41598-017-00797-2)
Supplement: Supplementary file 1 — Supplementary info [file 41598_2017_797_MOESM1_ESM.pdf]

Nanodiamonds as multi-purpose labels for microscopy

S. R. Hemelaar,[a]† P. de Boer,[b]† M. Chipaux,[a] W. Zuidema,[c] T. Hamoh, [a] F. Perona Martinez, [a] A. Nagl, [a] J.P. Hoogenboom,[c] B. N. G. Giepmans[b] and R. Schirhagl[a]

a. Groningen University, University Medical Center Groningen, Department of Biomedical Engineering, Antonius Deusinglaan 1, 9713 AW Groningen

b. Groningen University, University Medical Center Groningen, Department of Cell Biology, Antonius Deusinglaan 1, 9713 AW Groningen

c. Delft University of Technology, Dept. Imaging Physics, Lorentzweg 1, 2628 CJ Delft

Supplementary information

Quantification of diamonds in cells:

In order to quantify internalized particles we have developed a script to be used in the image analysis software FIJI (Fiji Is Just ImageJ, https://fiji.sc/). The analysis was divided into three phases: Cell Selection, Masking and Particle Analysis. During the first phase, the images were visually inspected and random cells were selected for the analysis. Cells which aggregates associated with the cell membrane were rejected to prevent false positive results. The images were composed of several slices (z-stacks), the cell's region was defined in all the three dimensions. In the horizontal plane, the selection considered an area containing only the cell of interest. In the height, the first and last slices containing the cell were identified. As a result, the first phase defines a volume that holds only the cell of interest. In the Masking phase, that volume is molded in order to resemble the shape of the cell. The image is converted to binary (using the Isodata algorithm to calculate the threshold)[1] then the cell's perimeter is detected in every slice. To find the inner volume of the cell, the program shrinks the cell's region in order to exclude the cell membrane from the analysis. The final step uses a special function of Fiji, which analyzes the particles found in a region. Applying this function to the masked image, it is possible to directly obtain the amount of particles (connected components) in the specified region. The performance of this process is set mainly by the parameter "threshold". The threshold is used to separate the background light from the signal emitted by the FNDs. Every pixel with intensity less than the threshold is assumed as background and deleted from the image (set as black) while every pixel with an intensity greater or equal than the threshold is assumed as part of a particle. To find an adequate value for this parameter, the image was visually inspected and different values were probed. Finally, the election was made in favor removing more background signal, but without deleting particles clearly identified, which showed no signal inside yeast cells without FNDs. As a result, the process provides the number of particles, which are found in every analyzed cell.

CL spectrum of NV_0

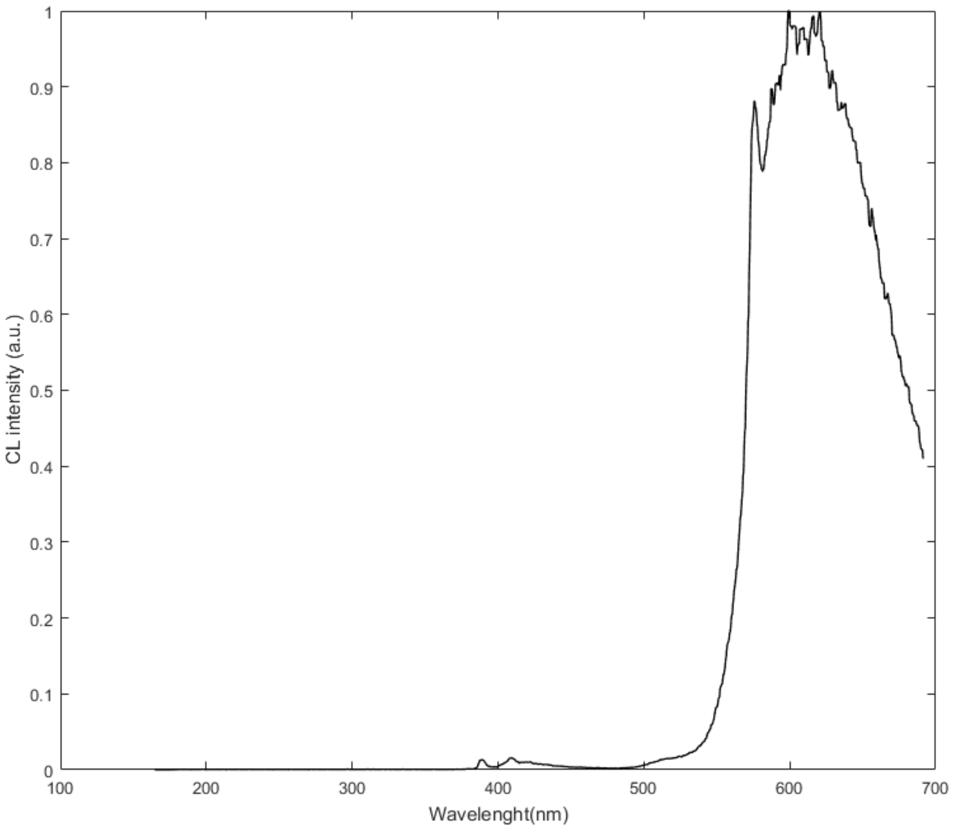

Figure S1: Cathodoluminescence spectrum from NV0 recorded while exciting with the electron beam

1. A. a Torrano, J. Blechinger, C. Osseforth, C. Argyo, A. Reller, T. Bein, J. Michaelis, C. Bräuchle, A fast analysis method to quantify nanoparticle uptake on a single cell level, Nanomedicine. 8 (2013) 1815–1828. doi:10.2217/nnm.12.178.
